# Relative Age Effect of Sport Academy Adolescents, a Physiological Evaluation

**DOI:** 10.3390/sports8010005

**Published:** 2020-01-07

**Authors:** Staffan Ek, Per Wollmer, Magnus K. Karlsson, Tomas Peterson, Ola Thorsson, M. Charlotte Olsson, Julia S. Malmborg, Magnus Dencker

**Affiliations:** 1 Department of Translational Medicine, Lund University, 221 00 Lund, Sweden; per.wollmer@med.lu.se (P.W.); ola.thorsson@med.lu.se (O.T.); Magnus.Dencker@skane.se (M.D.); 2 Department of Medical Imaging and Physiology, Skåne University Hospital, 205 02 Malmö, Sweden; 3 Clinical and Molecular Osteoporosis Research Unit, Department of Clinical Sciences and Orthopaedics, Lund University, Skåne University Hospital, 205 02 Malmö, Sweden; magnus.karlsson@med.lu.se; 4 Department of Sport Sciences, Malmö University, 205 02 Malmö, Sweden; tomas.peterson@mau.se; 5 The Rydberg Laboratory for Applied Sciences, Halmstad University, 301 18 Halmstad, Sweden; Charlotte.Olsson@hh.se (M.C.O.); julia.soderstrom_malmborg@hh.se (J.S.M.); 6 FoU Spenshult, Spenshult Research and Development Center, 301 18 Halmstad, Sweden

**Keywords:** relative age effect, fitness testing, physical maturation, talent selection

## Abstract

The relationship between birth quarter distribution and physiological characteristics related to athletic skills, in adolescent sport academy students has not been fully investigated. In a cross-sectional study, we recruited 86 boys and 52 girls aged 12–14 years during their first term at a sport academy school. We measured body size, cardiac size, pulmonary function, body composition, lower body power, cardiorespiratory fitness parameters, and running endurance by standard methods and analyzed these estimates in relation to birth quarter by ANOVA. Birth quarter distribution in our cohort was compared with birth quarter distribution in the same ages in the whole of Sweden and analyzed by logistic regression. The academy had an overrepresentation of students born in the first quartile of the year compared to those born in the last quartile (odds ratio 2.3 (95% CI: 1.1–4.7)). When comparing the physiological characteristics between birth quarters, uniformity is prominent since out of 26 performed physiological and anthropometric tests only four showed statistically significant group differences. We thus believe that the selection process to the sport academy favours athletes with higher chronological age, i.e., a so-called relative age effect is present.

## 1. Introduction

The relative age effect (RAE) is a well-observed and established phenomenon describing a bias evident in youth sport [1,2] and academia [3] in favour of subjects born earlier within an age group. It is especially prevalent in youth elite sport and consequently heavily debated within this field [4]. A common way to demonstrate the presence of RAE in a sport context is to analyse a population based upon birth date and participation [5,6,7,8,9]. Birth dates are translated into birth quarters of all born the same year. Individuals born in January to March may be compared to their younger peers born in October to December using January 1st as a cut-off date. If, when comparing the observed birth quarter distribution to the expected one based upon the population in general, older peers are overrepresented, a RAE is thought to be present.

In this study, we wanted to examine if a RAE is present at a Swedish sport academy where the students are specialized in diverse sports. To be able to study a possible RAE in greater depth, we also wanted to characterize the population in a variety of physiological ways, such as cardiorespiratory fitness, body power, body size, cardiac size, lung function, and body composition. Similar studies have previously been made on a selected study population of young elite level athletes, trying to characterize the cohort with respect to physical attributes [5,6,10,11] but to our knowledge none as comprehensive. We hypothesised that a RAE would be present in the selection process, with a skewed birth distribution towards being born early in the year, and that natural differences in physiological variables that occur within non-selected age groups, linked to performance, would be eliminated by the selection process. We further hypothesized that variables with no causal connection to performance but a stronger to chronological age (like bone mass) might still vary among birth quartiles.

## 2. Methods

### 2.1. Participants and Study Design

One hundred forty-seven students aged 12–14 years, who during two consecutive years were accepted to a sports academy, were asked to participate in this cross-sectional study when they started grade 7 (12–14 years of age). The sports academy is a community school situated in Malmö, Sweden, which offers education from seventh to ninth grade. All students are selected to the academy based upon sports merit and athletic skills trials, where the school claims to take into account not only current performance but also developmental potential. Several of the most popular sports in the area are represented. All physiological laboratory data were collected during one day of assessments, which took place in the span of September to February during the first year after being admitted to the academy in August, when the students were 12–14 years old. The performance tests took place in May during the same first year, i.e., when the students were 13–14 years old.

The two included age groups were born in two consecutive years which means that one age group were to complete the tests one year prior to the other one. All students gave their informed consent to participate, a letter of consent was signed by all participants and their parents (consistent with World Medical Association Declaration of Helsinki article 23 [12]) and the study was accepted by the Regional Ethical Review Board at Lund (2012/745). Out of 147 students in our cohort 138 (86 boys and 53 girls) completed a physiological evaluation in our laboratory. 

### 2.2. Measurement of Aerobic Fitness

Aerobic fitness was determined by a maximal exercise test, a test with gradually increased workload up to a limiting fatigue, that was performed on an electrically braked cycle ergometer (Rodby rhc, model RE 990, Rodby Innovation AB, Karlskoga, Sweden) with simultaneous analysis of respiratory gases [13]. Expired gas was sampled continuously via a mixing chamber and analysed for the concentration of O_2_ and CO_2_ (Sensor Medics 2900, SensorMedics Inc., Yorba Linda, CA, USA). Measurements were obtained every 20 s during 2 min at rest and during exercise to volitional exhaustion. All children, regardless of gender, fitness, height, and body mass, used the same protocol with an initial workload of 30 W and an increase of 15 watts per minute (1 W every 4 s). Maximum heart rate (HR_max_) and maximum respiratory exchange ratio (RER_max_) were recorded. The children had to fulfil one of three objective criteria; (1) max HR ≥ 185 beats/min, (2) RER ≥ 1.0, or (3) a levelling off or plateauing of VO_2PEAK_ (defined as an increase of oxygen uptake less than 150 mL during the last minute of exercise) [14,15]. The exercise test was considered acceptable if one of three objective criteria was fulfilled, and one or more of subjective criteria that indicated intense effort (e.g., hyperpnoea, facial flushing, or inability to keep adequate revolutions per min, or unwillingness to continue despite strong verbal encouragement).

### 2.3. Dual-Energy X-ray Absorptiometry (DXA)

A DXA scan (GE Lunar iDXA with with enCORE 13.60 software, General Electric, Madison, WI, USA) was used to estimate bone mineral content (BMC) and bone mineral density (BMD) excluding the head (Coefficient of variation (CV%): Total BMC 0.57% and Total BMD 0.59%) [16]. Total lean body mass (LBM) and total body fat mass (TBF) were quantified by DXA (CV%: Total Lean body mass 0.64% and total body fat mass 2.28%). Body fat was also calculated as a percentage of body mass (BF%). Trained research technicians performed all measurements and software analyses and also calibrated the DXA scan regularly with a phantom.

### 2.4. Pulmonary Function Testing

Pulmonary function tests, including Forced expiratory volume in 1 s (FEV_1_) and vital capacity (VC) were performed using Jaeger MasterScreener Body, Würzburg, Germany. All measurements were performed according to the recommendations of the American Thoracic Society/European Respiratory Society [17]. VC is presented as a percentage of predicted values based on gender and height, from Zapletal et al. equation [18].

### 2.5. Echocardiography

Three-dimensional (3D) imaging of the heart was performed using Philips IE 33 system (Koninklijke Philips N.V, Amsterdam, The Netherlands). The 3D images were obtained with the subject in the supine position over four cardiac cycles during a breath hold. Semiautomatic detection of the heart contour was performed after identifying specific heart points. A 3D endocardial volume of the LV was then produced from which LV volumes were calculated. Measurements of 3D LV volumes were performed (Q-Lab; Koninklijke Philips N.V, Amsterdam, The Netherlands) by one experienced echocardiographer. Left ventricular mass (LVM) was measured according to current guidelines [19], by two-dimensional guided M-mode from both parasternal short and long-axis views. Echocardiography measurements have been shown to be reasonably accurate and reproducible [19,20]. Measurements were made in duplicate from both views, by a single observer, and averaged. The results were adjusted for lean mass and body surface area.

### 2.6. Field Sport Performance Tests

Speed was measured by a 20 m straight speed test with infrared single-beam timing gates (MuscleLab; Ergotest Innovation, Porsgrunn, Norway) on an indoor track surface. Participants started 0.5 m behind the starting line and three trials were allowed, where the fastest time was recorded to the nearest 0.01 s for use in subsequent analyses.

Endurance was assessed with a 1000 m run, performed on a 200 m banked indoor track, measured with a stopwatch to the nearest second [21].

Lower body power was measured by the counter-movement jump (CMJ) with arm swing (CMJ-AS) [22] with an infrared mat (MuscleLab; Ergotest Innovation, Porsgrunn, Norway) [23]. Three jumps were performed, where the highest jump (to the nearest 0.1 cm) was used in subsequent analyses.

Grip strength was measured with a handgrip dynamometer (steel spring of 40 kg resistance; KERN Sohn GmbH, Balingen, Germany). The test was performed in the seated position with the arm resting on a table according to the Southampton protocol. Three attempts per hand were performed with the maximum value (recorded to the nearest 0.1 kg) used in subsequent analyses [24].

### 2.7. Data Analysis

Analyses were made in Statistica 12.0 (StatSoft Inc., Tulsa, OK, USA). Athletes were divided into four groups based on their date of birth. Accordingly, birth dates of groups quarter Q1, Q2, Q3, and Q4 were January–March, April–June, July–September, and October–December, respectively. The observed birth quarter affiliation groups of the accepted students were compared to corresponding birth rates in the general population during the same years in Sweden [25], which could be considered the expected birth quarter distribution. Since our cohort consisted of two consecutive age groups both birth years were accounted for and weighted based on its corresponding size in our population. The differences between observed and expected birth quarter distribution were analyzed using chi-squared distribution test. We report descriptive statistics as mean with standard deviations (SD). The equality of variances for studied variables was assessed by Levene’s test before comparing the four quartiles unadjusted with analysis of variance (ANOVA). The Shapiro–Wilks test was used as a test of normality. When testing for gender we used ANCOVA. To estimate the relative likelihood of students getting accepted to the school we used logistic regression by calculating the OR (95% CI) of selection (=1) in Q1, Q2, and Q3 compared with the referent category Q4 (OR = 1.00).

## 3. Results

A total of 138 participants had a complete dataset, drop-out was mainly due to difficulties to perform DXA scan (due to body taped fitness trackers being worn at the same time). The 138 participants participated in nine different sports, representing soccer (54, 39.1%), ice hockey (16, 11.6%), basketball (15, 10.9%), floorball (13, 9.4%), racket sports (12, 8.7%), athletics (6, 4.4%), figure skating (6, 4.4%), swimming (4, 2.9%), and diving (2, 1.5%), and with 10 doing more than one of already mentioned sports (7.3%). Out of the 138 participants, 102 (62 boys and 40 girls) also completed a set of field sports performance tests (countermovement jump, sprint, 1000 m run, and hand grip strength).

**Figure 1:**
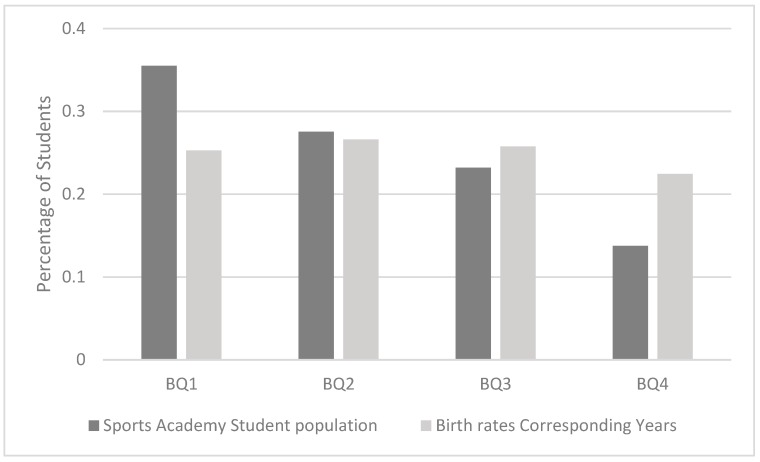
Expected (distribution in all of Sweden) and in our cohort observed birth quarter distribution (*p* = 0.01) Data is presented as proportions (%).

The sports academy had an over-representation of students born in the first quartile of the year (Odds ratio (OR) 2.3 (95% CI: 1.1–4.7)) when comparing children born January–March (Q1) to those born October–December (Q4). The ratios for Q2 and Q3 was 1.7 (95% CI: 0.8–3.5) respectively 1.5 (95% CI: 0.7–3.1). The birth quarter distribution of students selected to the school was proven to be skewed when comparing it to the general population of the same age in Sweden (*p* = 0.01) (Figure 1). Of the 29 tested dependent variables, only bone mineral content (BMC), bone mineral density (BMD), and total lean mass were statistically significantly different between the birth quarters (Table 1). Older students had, compared to younger students, higher BMC (Q1 vs. Q4 mean: 2.25 kg/1.97 kg (95% CI: 2.13–2.36 kg/1.86–2.08 kg)), higher BMD (Q1 vs. Q4 mean: 1.07 g·cm^−3^/0.99 g·cm^−3^ (95% CI: 1.04–1.09 g·cm^−3^/0.95–1.03 g·cm^−3^)), greater lean mass (OR Q1 vs. Q4 mean: 40.6 kg/36.2 kg (95% CI: 38.3–42.9/33.0–38.4 kg)) and faster 1000 m run test (OR Q1 vs. Q4 mean: 229 s/254 s (95% CI: 220–237 s/238–269 s)) (Table 2). Adjustment for gender did not change these results (all still being *p* < 0.05).

## 4. Discussion

By evaluating physiological characteristics related to athletic skills in adolescent sport academy school students and birth quarter distribution of acceptance to the school, we have shed light on this relationship in regard to the RAE. This study shows that students born in the beginning of the year may be favored in the selection process to a sport academy school, when the selection is based solely on sport merits. It was clear that athletes born early in the year were favoured to be selected compared to the ones born later. Even more dire is the difference in likelihood of being selected for the school when comparing first quarter to last (OR 2.3). As expected, this difference proved to be significant when it was set against the general population of the same age in Sweden. 

It is always problematic to select adolescents into a sport academy based upon physical performance [1,2,4]. When a cut-off date is used to determine the selection year, there will always be subtle but important age differences within the group. This categorization of pupils is used by the vast majority of school organizations, applying as well to sport academies in Sweden, using January 1st as the cut-off date. An up to one-year age difference, especially during the stages of puberty, can however affect physical [4,26] and performance [27,28] differences to a large extent. Hence, older athletes in the same year category have an edge over their relatively younger peers. A significant overrepresentation of athletes born in the first quartile has repeatedly been shown in various sports at a competitive level [1,2]. That relative age effect (RAE) pattern is widely established and recognized as an effect influencing selection systems in competitive youth sports all over the world [1,2,5,7,8,29,30,31]. The findings of our study are thus in agreement with previous studies and affirms our hypothesis that a RAE is present in the selection process, also to sports academy schools.

A secondary aim was to evaluate the importance of different physiological aspects in relation to an expected RAE. Our data indicate that significant differences of measured physical factors between birth quarters, with a weak connection to performance and a strong connection to chronological age, may still be prevalent after a hard selection process. 

Given the talent, or the ability to reach the elite level in sports, is evenly distributed among birth quarters and that the RAE has not played a part in the athletes’ development and acceptance into the sports academy, we would have expected to see a somewhat uniform distribution of accepted students from different birth quarters, reflecting the monthly birth count of the population in general. We would also expect to find significant differences in characteristics directly linked to sport performance, just as studies have done in non-selective populations according to birth date [32,33]. That was not the case, except for lean mass and the 1000 m run test. Since the group of athletes applying to the academy was not specifically tested for endurance and the population did not include any endurance athlete where the skill of 1000 m running is of great significance; i.e., there were no distance runners, orienteers or triathletes, one might speculate that this skill was not decisive in the selection process. Hence, no artificial uniformity of elite level skills between birth quarter groups was created regarding this particular characteristic. 

The fact that we did not find much difference between the groups exacerbates the picture of a hard selection with little consideration of physical maturity and relative age. We hypothesize that otherwise expected differences linked to sport performance would not be this absent if that were not the case. In nonselective schools, significant differences in physiological characteristics and fitness among their students are in contrast consistently found [32,33,34]. It is consistent with our hypothesis to imply that the significant differences actually found in BMD and BMC are more linked to age than to physiological performance [35]. It has also been shown in adolescents that muscle strength and performance are more depending on neuromuscular adaption than actual measured total lean mass [36]. Variables heavily linked to chronological age (or, for that part, athletic skills linked to age but not decisive in the selection process of a particular group), will not be eliminated by selection, just as variables linked neither to performance nor age.

## 5. Limitations

A limitation to this study is that we cannot establish if the skewed selection process with certainty can be attributed to the selection process of the school. Other reasons for it to appear could be plausible. A three-point integrated sociologic model has been proposed by Hancock and colleagues to explain the differences between birth quarters performance in sports, which chronological age cannot do on its own [37]. They argue that social agents have a vast effect on RAE in sports. This is thought to be achieved through sociological processes such as initial age advantages accumulating during the developmental course [38], early success leading to higher expectations from the surroundings (for example, coaches or parents) which is a self-fulfilling prophecy [39] and once expectations are placed upon an individual, that individual typically acts congruently with those expectations [40]. Mush and Grondin discussed the possibility of birth date effect being a result of gestational, climate, or environmental factors, depending on seasons (circannual), but sufficient evidence could not be found to support those ideas [2]. Instead, Veldhuizen and colleagues recently showed with data from a comprehensive prospective study, spanning over five years on Canadian adolescents being repeatedly examined with VO_2PEAK_ tests, that the RAE naturally prevalent in such a sample seemed to be accounted for by age alone [34].

## 6. Future Research

Although in line with previous research, this study relates to a specific school of ethnically and socially homogenous students. It is of interest to compare our results to schools attended by students with other backgrounds in western Europe as well as in schools with similar ambitions in other parts of the world. Another topic of interest would be if a RAE can be found in future sport results and indeed, participation in adult recreational sport of the students in this investigation. We intend to address both these questions in an international study and in a longitudinal study of the students in the present investigation, respectively.

## 7. Practical Considerations

Talent identification and subsequent selection during adolescence will always be problematic since large differences of maturity level exist and have been shown to have great influence on who makes the cut. Consideration should be given to individual maturity levels and relative chronological age, to not disadvantage athletes born late in the year. The staff in charge of the selection process should give more attention to technical and tactical skills as opposed physical characteristics. A system with birth quarter quotas could be considered as second phase intervention if other ways failed to come to terms with the problem.

## 8. Conclusions

In newly recruited students 12–14 years old in a sports academy, there was an overrepresentation of students born in the first quartile of the year compared to those born in the last quartile. Uniformity of relevant physiological characteristics between birth quarters was seen. The selection process to the sport academy thus seem to favor athletes with chronological higher age, i.e., a so-called relative age effect is present.

## Figures and Tables

**Table 1:** Anthropometry and physiology measures of pulmonary function, cardiorespiratory fitness body composition, cardiac and pulmonary function in 138 children born in different quartiles (Q) during the year. Data are presented as means and standard deviations. Statistically significant group differences in bold. Analysed with ANOVA.

Measurement	First Quarter	Second Quarter	Third Quarter	Fourth Quarter	*p*-Value
	N = 49	N = 38	N = 32	N = 19	
Height (cm)	165 ± 9.9	161 ± 9.0	164 ± 8.7	159 ± 6.3	0.09
Body Mass (kg)	53.6 ± 9.3	50.4 ± 9.3	52.1 ± 9.6	49.8 ± 6.7	0.30
BMI (kg/m^2^)	19.7 ± 2.0	19.2 ± 2.1	19.2 ± 2.4	19.6 ± 2.3	0.69
VO_2PEAK_ (L/min)	2.3 ± 0.68	2.2 ± 0.68	2.4 ± 0.59	1.9 ± 0.46	0.06
VO_2PEAKPKG_ (mL/(min·kg))	42.9 ± 11.4	43.8 ± 12.0	46.0 ± 7.2	38.8 ± 9.9	0.13
HR_max_ (bpm)	185 ± 12.8	183 ± 11.5	186 ± 11.5	186 ± 11.9	0.71
MAXW (W)	190 ± 36.5	181 ± 33.3	187 ± 39.1	165 ± 20.1	0.05
MAXWPKG (W/kg)	3.6 ± 0.53	3.7 ± 0.60	3.6 ± 0.57	3.3 ± 0.42	0.19
Systolic Blood Pressure (mmHg)	105 ± 9.8	103 ± 7.2	102 ± 9.7	102 ± 7.8	0.36
Diastolic Blood Pressure (mmHg)	56 ± 6.7	55 ± 6.6	57 ± 5.1	55 ± 6.9	0.65
Resting Heart Rate (bpm)	67 ± 9.1	68 ± 8.9	69 ± 9.8	69 ± 8.1	0.72
Average Percent Total Fat (%)	25.9 ± 6.7	24.6 ± 6.0	24.6 ± 5.3	27.4 ± 5.6	0.36
Fat Mass Total (kg)	11.7 ± 3.9	10.5 ± 3.8	11.1 ± 4.3	12.0 ± 4.0	0.44
Lean Mass Total (kg)	40.6 ± 8.0	36.9 ± 6.0	38.4 ± 6.4	36.2 ± 4.6	**0.03**
Total Bone Mineral Content (g)	2.25 ± 0.39	2.03 ± 0.30	2.13 ± 0.34	1.97 ± 0.23	**0.005**
Total Bone Mineral Densityl (g/cm^3^)	1.07 ± 0.087	1.01 ± 0.091	1.03 ± 0.090	0.99 ± 0.075	**0.005**
VC/Expected VC* (%)	102 ± 10	105 ± 10	99 ± 13	100 ± 13	0.17
FEV1.0/Expected FEV1.0* (%)	107 ± 14	106 ± 12	103 ± 15	107 ± 12	0.51
FEV1/VC*	87.9 ± 6.37	84.5 ± 7.09	86.9 ± 7.00	88.7 ± 5.67	0.07
DVLV/LM (mL/kg)	2.72 ± 0.46	2.88 ± 0.50	2.81 ± 0.52	2.79 ± 0.55	0.53
SVLV/LM (mL/kg)	1.03 ± 0.20	1.04 ± 0.21	1.07 ± 0.24	1.02 ± 0.22	0.82
LVM/LM (g/kg)	3.08 ± 0.61	3.31 ± 0.73	3.17 ± 0.74	2.97 ± 0.50	0.26

VO_2PEAK_—maximum oxygen uptake; VO_2PEAKPKG_—maximum oxygen uptake per minute per kg body mass; MAXW—maximal watt output; MAXWPKG—maximum watt output per kg body mass; VC—vital capacity; DVLV/LM—diastolic volume left ventricle divided with lean mass; SVLV/LM—systolic volume left ventricle divided with lean mass; LVM/LM—left ventricular mass divided with lean mass. * Expected based on height and Zaphletals equation (44).

**Table 2:** Performance in 102 children born in different quartiles (Q) during the year. Data are presented as means and standard deviations (SD). Statistically significant group differences are bolded.

Measurement	First Quarter	Second Quarter	Third Quarter	Fourth Quarter	*p*-Value
	N = 35	N = 27	N = 24	N =16	
20 m Sprint (s)	3.34 ± 0.18	3.38 ± 0.19	3.34 ± 0.17	3.46 ± 0.19	0.12
Countermovement jump (cm)	34.3 ± 5.3	33.4 ± 4.9	34.1 ± 5.9	31.9 ± 8.1	0.57
Maxgrip (kg)	30.3 ± 6.3	27.8 ± 5.5	30.4 ± 6.3	27.1 ± 6.3	0.16
1000 m (s)	228.7 ± 23.9	228.3 ± 26.4	223.3 ± 21.6	253.6 ± 29.7	**0.002**

Maxgrip—maximal handgrip pressure.
